# Using magnetoencephalography to examine word recognition, lateralization, and future language skills in 14-month-old infants

**DOI:** 10.1016/j.dcn.2020.100901

**Published:** 2020-12-17

**Authors:** Alexis N. Bosseler, Maggie Clarke, Kambiz Tavabi, Eric D. Larson, Daniel S. Hippe, Samu Taulu, Patricia K. Kuhl

**Affiliations:** a Institute for Learning & Brain Sciences, University of Washington, Box 357988, Seattle, WA, 98195, USA; b Department of Radiology, University of Washington, Box 354755, Seattle, WA, 98195, USA; c Department of Physics, University of Washington, Box 351560, Seattle, WA, 98195, USA; d Department of Speech and Hearing Sciences, University of Washington, Box 354875, Seattle, WA, 98195, USA

**Keywords:** Language, Right hemisphere, Attention, Infants, Magnetoencephalography

## Abstract

Word learning is a significant milestone in language acquisition. The second year of life marks a period of dramatic advances in infants’ expressive and receptive word-processing abilities. Studies show that in adulthood, language processing is left-hemisphere dominant. However, adults learning a second language activate *right*-hemisphere brain functions. In infancy, acquisition of a first language involves recruitment of bilateral brain networks, and strong left-hemisphere dominance emerges by the third year. In the current study we focus on 14-month-old infants in the earliest stages of word learning using infant magnetoencephalography (MEG) brain imagining to characterize neural activity in response to familiar and unfamiliar words. Specifically, we examine the relationship between right-hemisphere brain responses and prospective measures of vocabulary growth. As expected, MEG source modeling revealed a broadly distributed network in frontal, temporal and parietal cortex that distinguished word classes between 150–900 ms after word onset. Importantly, brain activity in the right frontal cortex in response to familiar words was highly correlated with vocabulary growth at 18, 21, 24, and 27 months. Specifically, higher activation to familiar words in the 150–300 ms interval was associated with faster vocabulary growth, reflecting processing efficiency, whereas higher activation to familiar words in the 600–900 ms interval was associated with slower vocabulary growth, reflecting cognitive effort. These findings inform research and theory on the involvement of right frontal cortex in specific cognitive processes and individual differences related to attention that may play an important role in the development of left-lateralized word processing.

## 1 Introduction

Acquiring a native language appears deceptively simple. Infants master complex details of its phonological, lexical, and syntactic properties by the age of 3 years, following the same developmental path regardless of culture (for reviews, see [Kuhl et al., 2008; Saffran et al., 2006]). Research over the last several decades has provided information about how infants achieve this task (Saffran et al., 2006; Kuhl, 2010), yet our understanding about the brain mechanisms that underlie language learning is far from complete. What these studies show is that children acquire language rapidly, exploiting the statistical and distributional patterns in environmental language input to learn (Kuhl et al., 2008; Goodsitt et al., 1993; Grieser and Kuhl, 1989; Saffran et al., 1996; Maye et al., 2002; Bosseler et al., 2014; Teinonen et al., 2009) and that constraints on perceptual and learning processes related to attention, especially those elicited through social interaction (Conboy and Kuhl, 2011; Kuhl et al., 2003), are critical to this process.

The neural mechanisms that underlie developmental mastery at each language level during development are key questions. Understanding language development poses a major scientific challenge that requires a description of not only the neural circuits that underlie the eventual left-hemisphere specialization observed in most adults, but also the brain mechanisms that are involved in and foster the initial phases of learning.

Substantial evidence, stemming from studies in children with brain lesions during the early period of language acquisition (Bates et al., 1997; Thal et al., 1991), and from research using electrophysiological (Molfese et al., 1991; Mills et al., 1997; Mills et al., 1993) and functional imaging (Dehaene-Lambertz et al., 2002) methods, suggests that a left-hemisphere dominance for language processing may not be present at birth but rather that it develops rapidly in infancy. Examination of the literature on infants and young children suggests that acquisition of a first language involves recruitment of bilateral brain networks, and, more surprisingly, that initial learning may depend on *right*-hemisphere brain functions (see also [Qi et al., 2015; Qi et al., 2019] for evidence with adults). For example, two prospective studies of infants with focal brain injury (Bates et al., 1997; Thal et al., 1991) report that word comprehension deficits are more pervasive in 10- to 17-month-old children with early unilateral *right*-hemisphere damage (see also [Aram and Eisele, 1994; Aram et al., 1987; Wulfeck et al., 1991)). These findings suggest that early language comprehension is a bilaterally distributed process, and that the right hemisphere plays a more vital role during the early stages of language learning than is currently reflected in developmental language theories (see also [Bates et al., 1995]).

The results of these studies raise interesting questions about interactions among brain systems underlying language learning and the cognitive functions associated with the right hemisphere, one of which is attentional control, in both children (e.g., (Thierry et al., 2003; Casey et al., 1997)) and adults (e.g., (Hampshire et al., 2020; Shallice et al., 2008b)). Infants’ ability to control attention has been shown to be associated with their early phonetic learning. Infants transition from “citizens of the world” discriminating all phonological contrasts at approximately 6 months of age to culture-bound listeners with good native and poor nonnative discrimination at approximately 12 months of age. The decline in discrimination of nonnative phonetic contrasts in language between 6 and 12 months of age has been linked to infants’ ability to inhibit attention in general cognitive tasks designed to measure the ability to control attentional processes (Diamond, 1990; Diamond et al., 1994). For example, 11-month-old infants with poorer nonnative speech discrimination show better performance on tasks requiring response inhibition (Conboy et al., 2008). The negative association between nonnative discrimination and cognitive control skills is interpreted as a reflection of infants’ increasing domain-general perceptual abilities to filter out irrelevant information allowing for more efficient attention to the native speech contrasts that allow them to distinguish between words (Diamond et al., 1994; Lalonde and Werker, 1995).

Another factor that will play a role in the current study is that the demonstration of learning efficiency at each level of language appears to set the stage for advances in learning at higher language levels. For example, infants’ increasing skill at discriminating native phonetic units improves their mastery of phonological rules, and predicts the speed of word learning and the mastery of sentence complexity to the age of 30 months, findings that hold across both behavioral (Kuhl et al., 2005; Tsao et al., 2004) and brain (Kuhl et al., 2008; Rivera-Gaxiola et al., 2005) measures. At the word level, studies on young children show that early efficiency in word processing is associated not only with faster vocabulary growth but also with long-term language and cognitive outcomes (Fernald et al., 2006; Marchman and Fernald, 2008).

Constraints on infants’ abilities to perceive and attend play an important role in learning efficiency. Infants cannot perceive all physical differences between speech sounds, nor do they learn all possible patterns represented in language input. What perceptual and attentional constraints “tune” infants to the critical components of their native language, and thereby promote efficient learning? In the current study, we focus on the right hemisphere areas that control attentional capacities and examine the role that activation in these brain areas in response to words plays in children’s initial language learning, and how these brain measures reflecting initial learning predict later language outcomes in young children.

### 1.1 The current study: word learning, attention, and the right hemisphere

Word learning is a significant milestone in language acquisition and the second year of life marks a period of dramatic advances in infants’ expressive and receptive word-processing abilities (Fernald et al., 2006; Fernald et al., 1998; Fernald et al., 2001; Zangl et al., 2005). To date, much of what is known about the neural processes involved in word processing stems from electrophysiological studies that utilize auditory event-related potentials (ERPs) to measure the electrical activity evoked as children listen to words (Mills et al., 1997; Mills et al., 1993; Conboy and Mills, 2006; Mills et al., 2005a; Mills et al., 2005b; Mills and Neville, 1997; Mills et al., 2004). Collectively, EEG studies in young children indicate that, during the early stages of word learning, brain activation during word processing is not only bilateral, but that there is an initial strong contribution of the right frontal brain region that attenuates as a function of age and language proficiency.

For example, an EEG study that examined the effect of word familiarity on 11-month-old infants provided evidence that right frontal attentional processes index the speed that familiar words are recognized (i.e., processing efficiency). Thierry, Vihman, and Roberts (Thierry et al., 2003) presented 11-month-old children with familiar and unfamiliar words and found that familiar words, but not unfamiliar words, elicited a significant response around 200 ms in the right hemisphere. Activity in this window has been associated with attentional shifting in adults (Näätäänen, 2001) and Thierry et al. suggested the hypothesis that activity in this early window reflects an involuntary shift of attention to familiar words and phonemes (see also [Hallé and Boysson-Bardies, 1994; Bosseler et al., 2013]) reflecting the speed or efficiency of processing when young children recognize and respond to familiar words.

Mills and colleagues documented the transition from predominately right/bilateral word processing to left-lateralized processing in typically developing infants and children between the ages of 13 and 20 months (Mills et al., 1997; Mills et al., 1993). Differences in the processing of familiar and unfamiliar words involve three distinct components exhibiting larger amplitudes to familiar than to unfamiliar words. The early and mid-latency components, occurring between approximately 200–400 ms and 400–600 ms post stimulus onset, are associated with word familiarity during development of efficient word processing (Mills et al., 2005a), with the earliest component reflecting an involuntary shift of attention to familiar words (Thierry et al., 2003). In contrast, the late latency component, with a latency of approximately 600–900 ms post stimulus, is thought to reflect slow, effortful processing, which recruits additional attentional resources to process familiar words (Mills et al., 2005a).

Mills and colleagues found that in the youngest and least proficient children significant ERP differences between familiar and unfamiliar words in the three time windows were not only broadly and bilaterally distributed, but larger over the right than the left hemisphere (see also (Thierry et al., 2003)). With increasingly efficient word processing, as indexed by age or productive vocabulary, the topography of the early and mid-latency responses to familiar and unfamiliar words shift from a broad, bilateral distribution to more focal ERPs over temporal and parietal regions of the left hemisphere. Critically, the late 600–900 ms effect, linked to attentional processing, disappears with increasingly efficient word processing. The transition in the pattern of response to familiar and unfamiliar words typically occurs by 20 months of age: as word processing becomes more efficient and children gain experience learning individual words, neural circuits undergo language specialization such that (a) linguistic computations become progressively more left lateralized (Mills et al., 1993; Mills et al., 2005a; Mills and Neville, 1997); and (b) the late right frontal response linked to attentional processing subsides. This pattern of results has been corroborated by studies of familiar and unfamiliar word processing in 19 to 22-month-old simultaneous bilingual children (Conboy and Mills, 2006), in late talkers up to age 30 months (Mills et al., 2005a), and in 18 to 30-month-old children with autism and typically developing controls (Kuhl et al., 2013). These data indicate that lateralization of brain activity is a dynamic process and is related to experience with the individual words (for discussion, see [Mills et al., 2005a]).

The presence of the right frontal response in all time windows for the youngest and least proficient children has been argued to relate to the allocation of attention (Mills et al., 1997; Mills et al., 2005a). The early latency left-lateralized shift and accompanying attenuation of the late latency response in older and more proficient children is thought to reflect increased processing efficiency co-occurring with neural specialization during development (for discussion, see [Mills et al., 2005a]). Collectively, these studies show that the pattern of event-elated potential (ERP) responses to familiar and unfamiliar words follows a specific developmental time course related to increasingly efficient word processing.

Functional imaging studies of adults acquiring a second language also show the contribution of right hemisphere attentional functions in the early stages of second language acquisition (Qi et al., 2015; Qi et al., 2019; Hosoda et al., 2013). Importantly, these studies show that individual differences in the degree of right frontal activation during the initial stage of learning is correlated with both the immediate attainment and long-term retention of a new language. For example, recent work by Qi and colleagues (Qi et al., 2019) found that individual activation differences in the right frontal cortex during language processing, measured before and after an intensive Mandarin language learning program, was strongly related to success in acquiring Mandarin as a second language. In the study, individuals with larger pre- training activation to lexical tones in the right inferior frontal gyrus (rIFG) showed better lexical tone discrimination and better Mandarin proficiency immediately after the training, as well as 90 days following training. Notably, activation in the rIFG was reduced post-training. The authors proposed that successful acquisition of a new language in adults requires increased rIFG during the initial learning and reduced rIFG activation for long-term retention of language abilities. The authors note that while learning Mandarin resulted in increased left IFG and left superior parietal lobule, activity in these regions did not relate to language learning outcomes. These results show levels of activation in the right fontal cortex during the initial stages of learning a new language is not only linked to greater proficiency for the newly acquired language (Hernandez et al., 2001;Stocco and Prat, 2014] see also, [Fletcher et al., 1999; Musso et al., 2003), but is predictive of long-term language learning success.

Importantly, there is now a substantial body of evidence using both behavioral and brain methods that show that early language measures predict subsequent milestones in language acquisition (Kuhl et al., 2008; Tsao et al., 2004; Rivera-Gaxiola et al., 2005; Kuhl, 2004; Kooijman et al., 2013). Here we focus on words, and there are brain data from studies using event-related potentials (ERPs) showing that individual differences in early word processing predict later language (Mills and Neville, 1997; Kuhl et al., 2013; Kooijman et al., 2013; Junge and Cutler, 2014; Junge et al., 2012a; Junge et al., 2010; Junge et al., 2012b). These data highlight the importance of the left hemisphere organization of language. However, recent data on adult language learning indicate that the *right* hemisphere is important during the earliest stages of the acquisition process (Qi et al., 2015; Qi et al., 2019; Hosoda et al., 2013). In the current study, our goal is to understand the role of early brain measures, particularly those focusing on the right hemisphere, on later language skills because (Kuhl et al., 2008) it will help us understand the mechanisms underlying language learning, and (Saffran et al., 2006) it may help us identify risk factors for children with language impairments such as autism, dyslexia, and SLI.

Previous studies show that language mastery at each level in the early phases of development, predict future language skills. At the phonetic level, multiple studies report that individual differences in measures of native and nonnative speech sound discrimination predict later language (Kuhl et al., 2008; Kuhl et al., 2003; Kuhl et al., 2005; Rivera-Gaxiola et al., 2005). At the lexical level, multiple ERP studies of word processing document a specific developmental time course transitioning from a broad and bilateral response with substantial right-hemisphere contributions to a more efficient focal and left-lateralized response related to age and language proficiency (Mills et al., 1997; Mills et al., 1993). Furthermore, individual differences in measures of word processing in children with autism have been shown to be related to later language skills (Kuhl et al., 2013).

Taken together, the results from both infant and adult studies support the view that attentional processes, mediated by the right frontal regions, are utilized during the earliest stage of language learning. In the current study, we hypothesize that the right frontal attentional networks are instrumental to the eventual left-lateralized processes that begin to occur in infancy. In particular, we argue that at the onset of language learning, these right hemisphere attentional mechanisms not only precede the eventual left hemisphere specialization, but also enable this process to occur by allocating attentional resources to the learning of language (see (Bosseler et al., 2013), for discussion). The goal of the present study is to investigate the relation between right frontal activity during word processing in 14-month old children and children’s subsequent language growth. On the basis of this work, we formulated two goals for the current study.

First, we examine the temporal and spatial properties of brain activation in response to familiar and unfamiliar words at 14 months, when young infants are on the cusp of producing their first words. Previous investigations of brain activation associated with familiar and unfamiliar words used the ERP method, which has relatively poor spatial resolution and cannot identify the specific origins of brain activity. Magnetoencephalography (MEG) is a brain imaging technique that allows the recorded changes in magnetic fields across the scalp to identify the sources of underlying neural activity, providing excellent spatial and temporal resolution. We employ MEG source modeling to more precisely identify the neural generators in the measurement windows described by earlier ERP studies of word processing in early (200–400 ms), mid (400–600 ms) and late (600–900 ms) measurement windows. We hypothesize that familiar and unfamiliar words will recruit neural generators in bilateral frontal, temporal, and parietal regions associated with word processing, consistent with previous ERP studies (Mills et al., 1997; Mills et al., 1993).

Second, we examine right frontal brain activity in infancy, in response to familiar words, in areas associated with attentional processes in adults, and relate those data to later language function measured behaviorally. On the basis of previous findings linking measures of familiar word processing to later language, and evidence that right-hemisphere frontal brain areas play a vital role during the early stages of language learning, we hypothesize that individual differences in the magnitude of right frontal brain activity in response to familiar words at 14 months of age are predictive of future language outcomes. Specifically, we posit that brain response magnitudes to familiar words in the right frontal cortex will be significantly associated with future vocabulary development assessed at the ages of 18, 21, 24, 27, and 30 months. We also predicted that attentional processes would differ as a function of measurement windows. The early window is related to automatic processing (signaling increased efficiency) (Thierry et al., 2003) and the late window with cognitive effort (signaling reduced efficiency) (Mills et al., 2005a). Consequently, larger response magnitudes to familiar words in the early measurement should be associated with faster subsequent vocabulary growth, whereas larger response magnitudes in the late window should predict slower vocabulary growth.

## 2 Methods

### 2.1 Participants

Twenty-seven typically developing (TD) English-learning infants were recruited for the study. The infants had no history of ear infections or other hearing difficulties, were born full term (39–42 weeks gestational age), and had normal birth weights (6–10 lbs). We obtained ethical approval from the University of Washington Human Subjects Division. The parents or legal guardians of all participants provided informed written consent as per the principles of the Declaration of Helsinki. We excluded data from five infants because of: later diagnosis of speech delay (n = 2), (SNR) excessive movement during data acquisition (n = 1), and equipment malfunction during recording (n = 1). The remaining twenty-two children (13 males) were on average 59.97 ± 0.58 (M ± SD) weeks old. Consistent with previous ERP word studies in young children (Conboy and Kuhl, 2011; Mills et al., 1993; Dehaene-Lambertz et al., 2002; Qi et al., 2019; Wulfeck et al., 1991), we created individualized lists of words based on parental ratings of a sample of 133 early-acquired monosyllabic nouns included in the CDI norming sample (Fletcher et al., 1999) and employed in a previous study (Mills et al., 1993).

### 2.2 Productive vocabulary assessment

Productive vocabulary was assessed when participants 18, 21, 24, 27 and 30 months of age using the MacArthur-Bates Communicative Development Inventory (CDI), specifically, the 680-word checklist, ‘Words and Sentences’ (Fenson et al., 1993; Fenson et al., 2006). Using the CDI, parents reported the number of words produced by their child based on the 680-word checklist section of the CDI when their child reached the target age.

### 2.3 MEG data acquisition

Infants were prepared for testing outside the magnetically shielded room (MSR) while a research assistant entertained them. A 3D position monitoring system (Polhemus, Colhester, VT) was used to record the locations of head position indicator (HPI) coils, cardinal (nasion, left/right preauricular) anatomical landmarks, and additional points (> 100) covering the scalp. Participants were placed in an infant seat made for use in the MEG scanner to record brain activity, see Fig. 1 in SI. The child’s head was centered and positioned as high as possible relative to the MEG dewar, using foam cushions and padding. All infants were awake and alert during recordings. A female research assistant entertained the infant using silent toys, and a silent video in the background throughout the recording session. We recorded MEG signals with a 306-channel whole-scalp MEG system (VectorView™, Elekta Neuromag Oy, Helsinki, Finland) within a magnetically shielded room (MSR) at the Institute for Learning & Brain Sciences, University of Washington, Seattle, WA. Neuromagnetic data was sampled at 1 kHz with a pass-band of 0.01–300 Hz. During MEG recordings signals from the HPI coils were used to continuously track the child’s head position relative to the MEG sensors.

**Fig. 1:**
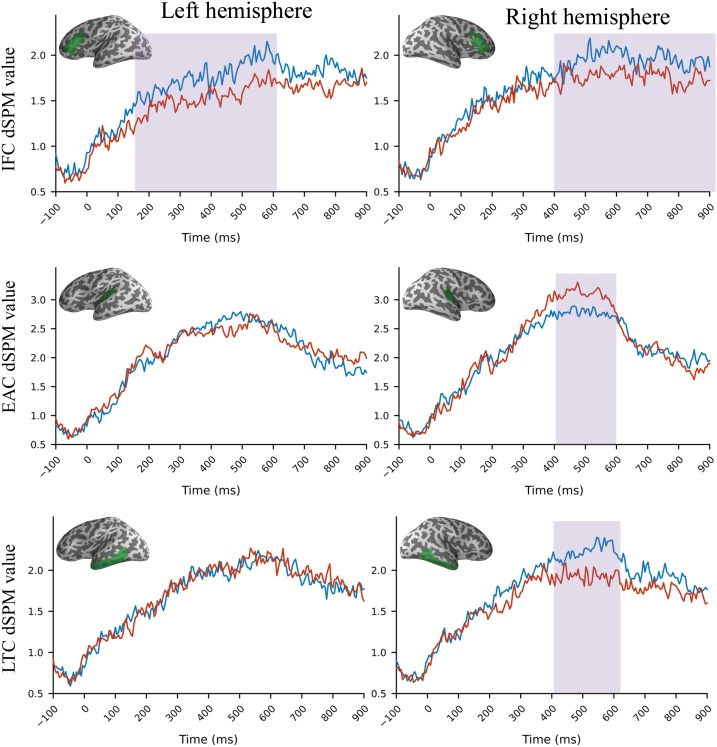
Grand averaged source waveforms in the left (left panel) and right (right panel) hemispheres for each selected anatomical label: Inferior Frontal Cortex (IFC), Early Auditory Cortex (EAC), Lateral temporal Cortex (LTC). Figures show the grand averaged dSPM values. Shaded areas reflect significant differences between familiar (blue line) and unfamiliar (red line) words (see Fig. 2).

### 2.4 Data reduction and analysis

MEG data signal processing was carried out using MNE-Python (Gramfort et al., 2013; Gramfort et al., 2014). To suppress signal artifacts from external sources, the data were processed using temporal signal space separation (Taulu and Kajola, 2005); correlation limit 0.98, window duration 4 s). MaxFilter was used to estimate continuous head position from the HPI coil positions, and the resulting head position data were used to perform movement compensation in MNE-Python. The position [0,0,40] mm in the MEG device coordinate frame was used as the target head position across subjects to facilitate cross-subject sensor-space comparisons. Line noise (60 Hz and harmonics) and HPI coil frequencies were removed from the data using windowed time-varying amplitude estimates (similar to -hpifit in MaxFilter), and data were then low-passed at 80 Hz. Signal space projection (Hämäläinen, 1995) was used to suppress the cardiac signal in the MEG data by estimating two orthogonal vectors capturing the spatial structure of heartbeats based on the time epochs encompassing the QRS complex, which was identified in time based on a maximally responsive gradiometer sensor in each subject. To optimize SNR and eliminate artifacts, trials were rejected where: (a) MEG sensors exceeded thresholds determined on a per-subject basis automatically using the autoreject package (Jas et al., 2017); (b) fewer than three HPI coils were locatable for at least 1 s; or (c) rotational and translational velocity exceeded 20 deg/s or 1 cm/s during the trial. The remaining single-trial data were averaged to create ERF datasets for each word type using one-second epochs (0.1 s pre-stimulus) of neuromagnetic data around each stimulus. To correct DC offset we subtracted the mean value of amplitudes across MEG sensors in the pre-stimulus interval from individual sensor values in the post-stimulus period. Before MEG source modeling the number of trials for each stimulus type was equalized to avoid averaging bias across different conditions.

### 2.5 MEG source localization

To estimate the location of neural generators underlying MEG signals, each subject’s anatomical landmarks and additional scalp points were used with an iterative nearest-point algorithm to rescale a surrogate 14-month-old subject MRI to match the subject’s head shape. FreeSurfer was used to extract the inner skull surface (watershed algorithm) and the gray/white cortical surface segmented from the surrogate MRI (Dale et al., 1999). A one-layer conductor model based on the rescaled inner skull surface was constructed for forward modeling (Hämäläinen and Sarvas, 1989). For each subject, the source space consisted of 4098 dipoles per hemisphere located along the gray/white matter boundary, evenly spatially distributed based on the correspondence of a recursively subdivided (six times; “oct6″) octahedron with the FreeSurfer spherical surface for each subject. Because surrogate head models and source spaces were used for each subject, source orientations were unconstrained (free orientation). Baseline noise covariance was estimated from 100 ms prior to the initial onset and spatial whitening was performed using the estimated noise covariance matrix based on the “shrunk” advanced estimator with automatic regularization (Engemann and Gramfort, 2015). Dipolar currents were estimated from the MEG sensor data using an anatomically constrained minimum-norm linear estimation approach to obtain dSPM values (Hämäläinen and Sarvas, 1989; Dale and Sereno, 1993; Hämäläinen and Ilmoniemi, 1994) using the magnitude of the vector estimate at each source location. For each time window of interest: 150–350 ms (early), 400–600 ms (middle), and 600–900 ms (late) we further assumed generators in the bilateral frontal, temporal and parietal regions subselected from on the reduced version human connectome project (WU-Minn HCP data) multimodal parcellation consisting of 46 cortical labels based on the (HCPMMP1; see supplemental information 3 from [Glasser et al., 2016]).

### 2.6 Statistical analysis

Linear mixed effect models (LMMs) were used to test for differences between response magnitudes to familiar and unfamiliar words and to compare those differences among regions and hemispheres. The models used a random intercept to account for repeated measures and fixed effect terms to represent average differences between familiar and unfamiliar words (indicated using the Δ symbol in tables and text) and differences between regions and hemispheres. The response magnitudes were log-transformed before modeling to reduce right skewness.

LMMs were also used for growth curve modeling of vocabulary growth over follow-up visits at 18, 21, 24, and 27 months and testing whether ERF responses to familiar words at 14 months in the IFC was predictive of vocabulary growth at 18–27 months. Vocabulary growth was transformed before modeling to account for right-skewness, saturation of the word list, and non-linearity. Vocabulary growth was first divided by its maximum value to change it to a proportion; the arcsin-transformation $\left({\text{sin}}^{-1}\sqrt{x}\right)$, a common variance-stabilizing transformation for proportions, was then applied; lastly, the result was standardized to have 0 mean and unit standard deviation. Spearman’s rank correlation coefficient was also used to explore these associations at individual follow-up visits.

All statistical calculations were conducted with the statistical computing language R (version 3.1.1; R Foundation for Statistical Computing, Vienna, Austria). Throughout, two-sided tests were used, with statistical significance defined as *P* <  0.05, without adjustment for multiple comparisons.

## 3 Results

### 3.1 ROI analysis: event related fields in response to familiar vs. unfamiliar words

Fig. 1 shows grand-averaged source level waveforms for familiar and unfamiliar words in the left and right hemispheres for each selected region of interest (ROI): Inferior Frontal Cortex (IFC), Early Auditory Cortex (EAC), and Lateral Temporal Cortex (LTC). The figure shows high signal-to-noise ratio in response magnitudes for familiar and unfamiliar words at each ROI that are broadly and bilaterally distributed. By visual inspection, differences between familiar and unfamiliar words emerged by 150 ms and are sustained across the entire epoch; they differ however in the temporal domain as a function hemisphere and anatomical label.

**Fig. 2:**
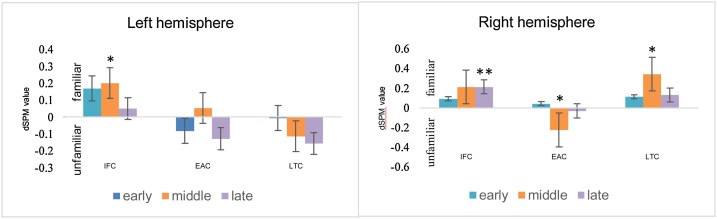
Grand averaged dSPM difference values for familiar–unfamiliar words in the left (left panel) and right (right panel) hemispheres for the selected anatomical label: Inferior Frontal Cortex (IFC), Early Auditory Cortex (EAC), Lateral Temporal Cortex (LTC). Difference values are shown for the early (blue bar), middle (green bar) and late (yellow bar) latency windows. Positive values indicate larger response magnitudes to familiar vs. unfamiliar words, whereas negative values indicate larger response magnitudes to unfamiliar vs. familiar words, *P <  0.01, ** *P* <  0.05, calculations based on Kenward-Roger approximation.

Fig. 2 shows the mean differences between response magnitudes for familiar vs. unfamiliar words in each ROI for each measurement window (See Supplemental Tables 1–3 for all individual modeling results). The difference in mean magnitude between word type x ROI x hemisphere were examined with dynamic statistical parametric mapping (dSPM) (Dale et al., 2000) within the early (150−350 ms), middle (350−600 ms) and late (600−900 ms) measurement windows. As shown in the figure, these windows captured most of the magnitude differences between familiar and unfamiliar words and is consistent with measurement windows used in previous ERPs studies (Mills et al., 1997; Mills et al., 1993; Conboy and Mills, 2006). Linear mixed effect models in the early measurement window (Supplemental Table 1), indicated that the difference in response magnitudes to familiar and unfamiliar words was not significant when averaged across all regions and hemispheres (Δ = 0.06; 95 % CI, −0.03 to 0.15; *P* = 0.16). Planned comparisons for each ROI were conducted based on previous EEG research reporting hemispheric differences, revealing a trend toward greater response magnitudes for familiar words in the left inferior frontal cortex (IFC) (Δ = 0.14; 95 % CI, −0.01 to 0.28; *P* = 0.061) and no statistically significant difference in the other ROI *(P* > 0.11 for each) (Supplemental Table 1). In the middle measurement window, the difference in response magnitudes to familiar and unfamiliar words was not significant when averaged across all regions and hemispheres (*P* =  0.41, Supplemental Table 2, Fig. 2b). Planned comparisons indicated that the magnitude of the response to *familiar* words was significantly larger than unfamiliar words in left IFC (Δ = 0.16; 95 % CI, 0.00 to 0.31; *P* = 0.043) and right Lateral Temporal cortex (Δ = 0.20; 95 % CI, 0.05 to 0.35; *P* = 0.011). In contrast, the magnitude of the response to *unfamiliar* words was significantly larger than familiar words in right early auditory cortex (EAC) (Δ = −0.17; 95 % CI, −0.33 to −0.02; *P* = 0.028). In the late measurement window, the left and right hemispheres differed significantly in terms of the magnitude of the response to familiar and unfamiliar words (Δ = −0.02 vs. 0.08; *P* = 0.031) (Fig. 2 and Supplemental Table 3). When examined by ROI, the response magnitude between familiar and unfamiliar words was significantly different in the right IFC (Δ = 0.19; 95 % CI, 0.05 to 0.34; *P* = 0.01), but not in any other individual ROI (*P* > 0.31 for each).

### 3.2 Relationships between the right frontal brain activity to familiar words and later language

The second goal of the current study was to examine the relationship between the right frontal brain activity to familiar words and productive vocabulary as measured using the MacArthur-Bates Communicative Development Inventories (CDI, see Materials and Methods) – Words and Sentences (Fenson et al., 1993). Eighteen families completed the CDI survey at 18, 21, and 24 months and 17 completed CDIs at 27 and 30 months. The median (IQR) vocabulary growth at each target age was 102 (50–211), 201 (119–340), 336 (212–495), 481 (371–597), and 600 (512–665), respectively. The 30-month CDI time point was not included in the growth curve analysis because 6 of 17 children (35 %) were within 90 % of the upper limit of the word lists, indicating ceiling effects. When examining each CDI score at each age separately, the response magnitudes to familiar words in the right IFC in the early window were correlated with a larger vocabulary from 18 months (r = 0.43; *P* = 0.078) to 30 months (r = 0.73; *P* = 0.001). In contrast, there were no significant correlations between vocabulary and response magnitudes in the middle latency window (r = 0.04 to 0.24; *P* > 0.36) and the late latency window (r = −0.28 to −0.12; *P* > 0.13).

**Fig. 3:**
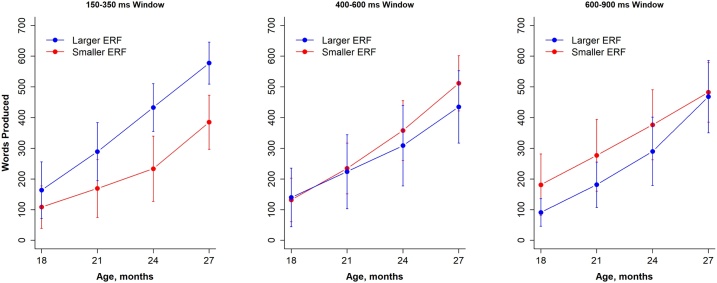
A median split of infants’ response magnitudes (blue = larger magnitude, red = smaller magnitude) in response to familiar words in the right IFC in 14-month-old infants is shown along with the averaged growth in words produced between 14 and 30 months of age. Longitudinal growth curve functions are shown for the early window (left panel), mid window (middle panel) and late window (right panel). The error bars are 95 % confidence intervals. As shown in Table 1, there was a significant positive relationship between vocabulary growth and ERF response magnitude in the early measurement window (higher response magnitudes above the average model prediction out to 27 months of age, *P* = 0.001) and a trend towards a negative relationship in late window (higher response magnitudes below the average model prediction, *P* = 0.077).

Growth curve models for productive vocabulary growth were then utilized to increase statistical power over any individual CDI follow-up time point. Individual vocabulary trajectories and the average prediction are shown in Fig. 3. Modeling results are shown in Table 1. The response magnitudes to familiar words in the right IFC during the early window was significantly correlated with larger vocabulary growth over time (β = 0.51; 95 % CI, 0.24 to 0.78; *P* = 0.001). There was a trend towards a negative relationship between response magnitudes in the late window and vocabulary growth (β = −0.30; 95 % CI, −0.63 to 0.04; *P* = 0.077) and there was no apparent relationship during the middle window (β = 0.00; 95 % CI, −0.36 to 0.36; *P* = 0.99).

**Table 1:** Associations between response magnitudes in the IFC to familiar words at 14 months and growth of vocabulary across 18-, 21-, 24-, and 27-month CDI measures.

Region	Window	Β^a^	(95 % CI)	P
Right IFC	150–300 ms	0.51	(0.24, 0.78)	0.001
	400–600 ms	0.00	(−0.36, 0.36)	0.99
	600–900 ms	−0.30	(−0.63, 0.04)	0.077

Left IFC	150–300 ms	0.10	(−0.26, 0.46)	0.56
	400–600 ms	−0.05	(−0.42, 0.31)	0.76
	600–900 ms	−0.03	(−0.39, 0.34)	0.87

a Mean change in vocabulary growth, after data-transformation (see Statistical Analysis section).

## 4 Discussion

Right-hemisphere contributions have not been emphasized in current theories of initial language acquisition. However, the right hemisphere is often reported as the dominant brain hemisphere activated in young children as they listen to familiar words, and the right hemisphere has been associated with learning and attention control (Hampshire et al., 2020; Shallice et al., 2008a; Shallice et al., 2008b). The right frontal cortex is also active in adults as they process a newly learned second language and is positively correlated with performance in that language (Qi et al., 2015; Qi et al., 2019; Hosoda et al., 2013). These studies and other previous work reveal a complex picture that places the right hemisphere at the center of initial language learning, raising interesting questions about interactions among brain systems associated with the right hemisphere that particularly highlight the role that attention plays in initial language learning.

The combination of excellent temporal and spatial resolution provided by MEG source modeling allows more precise identification of the neural generators in the measurement windows described by earlier ERP studies of word processing. To our knowledge this is the first study that MEG brain imaging has been used in a study of 14-month-old children as they listen to familiar and unfamiliar words. It is also the first study to examine right frontal brain activity with respect to prospective measures of vocabulary growth in young children. We hypothesized that familiar and unfamiliar words would recruit neural generators in bilateral frontal, temporal, and parietal regions associated with word processing, consistent with previous ERP studies of children early in the second year of life (Mills et al., 1997; Mills et al., 1993); that is, significantly stronger response to familiar words were expected in three time windows that are not only broadly and bilaterally distributed, but larger over the right than the left hemisphere.

Using a familiar–unfamiliar word paradigm, we first examined differences in response magnitudes of familiar and unfamiliar words in early, middle and late time windows. We found that differences between familiar and unfamiliar words emerged by 150 ms, with overall difference magnitudes larger to familiar versus unfamiliar words. These differences were broadly and bilaterally distributed over frontal, temporal, and parietal regions, and were more likely to be significant over the right hemisphere.

In addition to the similarities, there were differences in the results of the current MEG study and earlier EEG studies of word processing. Previous EEG studies consistently report larger familiar versus unfamiliar responses in the right versus left hemisphere in young children across all time windows. Our MEG analysis, however, reveals that in the early time window, significantly larger response magnitudes occurred to familiar words in the *left*, but not *right*, frontal cortex, and we speculate that this activity may be related to motor planning.

The left frontal cortex houses Broca’s area, which is thought to support speech motor planning. Using MEG, Kuhl and colleagues (Kuhl et al., 2014) previously showed that in 7-month-old infants both native and nonnative syllables activate the left IFC to an equal extent. However, by 11 months, nonnative discrimination activates Broca’s area to a greater degree, a finding identical to that shown by adult listeners (Callan et al., 2004; Callan et al., 2003; Dehaene et al., 1997; Golestani and Zatorre, 2004; Menning et al., 2002; Nakai et al., 1999; Wang et al., 2003). Kuhl and colleagues (2014) interpret these findings within an analysis-by-synthesis model, emphasizing that as infants listen to speech, they generate nascent motor models of speech signals internally, based on their experience both hearing and producing speech. Stronger activation in the left inferior frontal cortex in response to nonnative speech at 11 months may reflect infants lack of experience with nonnative speech, and a corresponding difficulty in generating internal motor models as well as increased processing demands when listening to nonnative speech. In line with this previous study, the current results allow us to speculate that, at 14 months, when infants are just beginning to produce their first words and processing demands for familiar words remains high, children generate an internal model of the speech motor plans as they listen to familiar words, which may help to strengthen and refine their speech-motor representations.

The results in the middle and late time windows more closely resembled the broadly distributed and largely right-lateralized effect reported in previous EEG studies. However, the high spatial resolution of MEG showed variability in the relative response magnitudes of familiar and unfamiliar words within the auditory cortex during the middle time window. Specifically, the response is significantly greater for familiar than unfamiliar words in the right temporal cortex, while the response is significantly greater for unfamiliar words in the right early auditory cortex. This pattern of results is consistent with a current view of language processing, which proposes large-scale networks that consist of multiple brain areas, each with specialized functions. This language network, mediated by a “dual-stream” of connections between frontal, temporal and parietal lobes (Rauschecker, 1998; Hickok and Poeppel, 2007), is involved in either mapping sounds onto meaning (the “ventral stream”) or mapping sounds onto articulatory representations (the “dorsal stream”). The ventral stream consists of the superior and middle portions of the temporal lobe and regions of the inferior frontal lobe. Together these regions are believed to mediate auditory comprehension by transforming auditory, or phonological, input to the mental lexicon, processes that are essential to determining the meaning of individual words and phrases (e.g. Hickok and Poeppel, 2007; Skeide and Friederici, 2016). The early auditory cortex, auditory association area, and lateral temporal cortex are a part of the ventral stream, yet serve different functions. The auditory association area, or Wernicke’s area, and the temporal pole region of lateral temporal cortex are considered to be the core of semantic processing (Binder et al., 1997). Our results show that in these semantic regions, response magnitudes were larger to familiar versus unfamiliar words, suggesting that infants are accessing the meanings of familiar words. In contrast, the larger response magnitudes to unfamiliar words in the early auditory cortex, which is comprised of BA41 and BA42 and involved in phonological word form detection, indicates a greater contribution of the early auditory cortex to process unfamiliar word forms. The larger right-hemisphere response magnitude to unfamiliar words in this region is consistent with recent results reported in 6- to 8-year-old children, in which unfamiliar words elicit larger response amplitudes in right superior temporal cortex compared with familiar words (Nora et al., 2017).

Our second goal was to examine individual differences in response magnitude of the right frontal brain activity in response to familiar words at 14 months of age to determine whether early individual differences can be linked to children’s future language outcomes. Specifically, we hypothesized that response magnitudes to familiar words in right frontal cortex would predict vocabulary growth between the ages of 18 and 30 months. Furthermore, we predicted that the direction of association would differ based on the attentional processes associated with the early (processing efficiency) and late (cognitive effort) measurement windows.

Our results support these hypotheses: response magnitudes to familiar words in the early and late time windows at 14 months of age both predict future language growth, but in opposite directions. We found that larger response magnitudes to familiar words in the early window predict faster vocabulary growth, whereas larger response magnitudes to familiar words in the late window predict slower vocabulary growth. However, it should be noted that while in our analysis the early window result is statistically significant, the late window result is supported by a statistical trend that did not quite reach statistical significance (p = 0.077), so a larger study may help confirm the interesting late window finding. The negative correlation between vocabulary growth and larger response magnitudes in the late time window are consistent with results of previous ERP studies, which show that larger responses to familiar words in the right frontal region are observed only in typically developing children between the ages of 13–17 months and in 20-month-old late talkers, but not in 13- to 17-month-old children with large vocabularies or typically developing 20-month-old children (Mills et al., 1997; Mills et al., 1993). Mills, Conboy and Paton (Mills et al., 2005a) hypothesized that activity in this later window over right frontal electrodes may reflect slow, effortful processing in which additional attentional resources are needed to process familiar words. Thus, larger response magnitudes to familiar words in this late window may reflect the protracted allocation of attentional resources to familiar words which attenuates as the child gains experience with individual words (see Mills et al., 2005a).

Several functional imaging studies with adult participants indicate that right IFC contributes to the acquisition of a second language, and show a positive correlation between the strength of the response in this region when listening to their second language and their proficiency with that language. Although these fMRI studies lack temporal information, the results are consistent with the results of a previous EEG study, which show that familiar words elicit a larger response at around 200 ms over right frontal electrode sites compared with unfamiliar words in 11-month-old infants. The authors interpreted this larger response as an index of the attentional involvement related to processing efficiency. Specifically, they argued that the larger 200 ms response reflects the capture of infant attention to and recognition of familiar words early in the processing stream.

Our current results advance previous EEG findings by identifying the brain areas involved in this critical process. We demonstrate, for the first time, that the magnitude of response to familiar words at 14 months in both the early and late time windows is predictive of the rate at which new words are acquired up to 27 months of age. These findings lead to the working hypothesis that attentional processes have powerful effects in learning new linguistic information and contribute to processing efficiency. We believe that the correlations between response magnitudes to familiar words at 14 months and later productive vocabulary provide support for the critical role of right-hemisphere function in the development of left-hemisphere language specialization in the brain.

Given the dynamic nature of language development, it is important to acknowledge that the results of the current study are based on brain activity measured at a single time point in development and therefore do not allow us to describe the ongoing impact of right frontal activity and subsequent brain activity as language develops. Future research would benefit from other sampling approaches. For example, it would be of theoretical interest to evaluate brain activity to words in the same children at several points in development to observe whether children with greater right frontal activity at the earliest stages of word learning are faster to show left hemisphere specialization.

Although the effects reported in this study are robust, our results are based on families with relatively high average socioeconomic status (SES) despite the fact that the families came from a variety of SES backgrounds. Future studies would benefit from recruiting a larger and more diverse sample of children to show whether results vary with SES and cultural backgrounds.

## 5 Conclusions

MEG results from 14-month-old infants provide strong evidence that right frontal brain regions play an important role during the earliest phases of word learning. MEG source modeling revealed a broadly distributed network in frontal, temporal and parietal cortex that distinguished word classes between 150–900 ms after word onset. Importantly, brain activity in the right frontal cortex in response to familiar words was highly correlated with vocabulary growth at 18, 21, 24, and 27 months. These findings cannot be attributed to differences in the left hemisphere contribution, because left hemisphere activity at this early stage of language learning was not predictive of subsequent vocabulary growth. These results inform theory on the involvement of right frontal cortex in specific cognitive processes, and also suggest that individual differences related to attention may play a more important role in vocabulary acquisition than is reflected in current theories of language acquisition.

## Classification

Major: Biological Sciences

Minor: Physiological and Cognitive Sciences

## Significance Statement

Word learning is a key milestone in infant language acquisition. In adulthood, language processing is left-hemisphere dominant. However, there is evidence to suggest that initial learning of new language material in adults may depend on *right*-hemisphere (RH) brain functions. We used magnetoencephalography (MEG) in 14-month-olds on the cusp of word learning to characterize neural activity in response to familiar and unfamiliar words, and examined the magnitude of brain responses in right frontal cortex as a predictor of later vocabulary growth. MEG data analysis of RH areas related to attention demonstrated that infants’ responses to familiar words at 14 months predicted the rate at which infants acquired new words at 18, 21, 24, and 27 months. We argue that RH activation reflects processing efficiency for familiar words.

## Author contributions

K.T., A.N.B., and P.K.K. designed research; K.T. performed research; A.N.B, M.C., E.D.L., and S.T. analyzed data; A.N.B. and D.S.H. performed statistical analysis; A.N.B., E.L., D.S.H., and P.K.K. wrote the paper.

## Declaration of Competing Interest

The authors declare that they have no known competing financial interests or personal relationships that could have appeared to influence the work reported in this paper.
