# Supplementary material for: Using magnetoencephalography to examine word recognition, lateralization, and future language skills in 14-month-old infants
Source: Dev Cogn Neurosci. 2020 Dec 17;47:100901. doi: 10.1016/j.dcn.2020.100901 (PMC7773883; doi:10.1016/j.dcn.2020.100901)
Supplement: Supplementary file 1 [file mmc1.docx]

**Supplemental Table 1.** Comparison of ERF responses to known and unknown words by region and hemisphere in the early measurement window.

|  | **150-350 ms Window** | | | | | |
| --- | --- | --- | --- | --- | --- | --- |
|  |  |  |  |  |  |  |
|  | **Word Type*** | |  | **Difference†** | | |
|  | **Known** | **Unknown** |  | **Δ** | **(95% CI)** | **P-value†** |
| **Overall** | 1.9 ± 0.7 | 1.8 ± 0.7 |  | 0.06 | (-0.03, 0.15) | 0.16 |
|  |  |  |  |  |  |  |
| **Region** |  |  |  |  |  |  |
| EAC | 2.2 ± 1.1 | 2.2 ± 1.0 |  | -0.02 | (-0.13, 0.09) | 0.73 |
| IFC | 1.7 ± 0.4 | 1.5 ± 0.4 |  | 0.10 | (-0.02, 0.21) | 0.091 |
| IFPC | 1.9 ± 0.6 | 1.7 ± 0.6 |  | 0.10 | (-0.02, 0.21) | 0.096 |
| LTC | 1.8 ± 0.5 | 1.6 ± 0.4 |  | 0.08 | (-0.04, 0.19) | 0.17 |
| (p-value)‡ |  |  |  |  |  | 0.18 |
|  |  |  |  |  |  |  |
| **Hemisphere** |  |  |  |  |  |  |
| Left | 1.9 ± 0.7 | 1.7 ± 0.7 |  | 0.07 | (-0.03, 0.16) | 0.18 |
| Right | 1.9 ± 0.8 | 1.8 ± 0.7 |  | 0.06 | (-0.04, 0.16) | 0.22 |
| (p-value)‡ |  |  |  |  |  | 0.89 |
|  |  |  |  |  |  |  |
| **Region-Hemisphere** |  |  |  |  |  |  |
| Left EAC | 2.2 ± 1.1 | 2.3 ± 1.1 |  | -0.04 | (-0.18, 0.11) | 0.62 |
| Right EAC | 2.2 ± 1.2 | 2.2 ± 0.9 |  | 0.00 | (-0.15, 0.14) | 0.95 |
| Left IFC | 1.7 ± 0.5 | 1.4 ± 0.3 |  | 0.14 | (-0.01, 0.28) | 0.061 |
| Right IFC | 1.7 ± 0.4 | 1.6 ± 0.5 |  | 0.06 | (-0.08, 0.20) | 0.41 |
| Left IFPC | 1.9 ± 0.6 | 1.6 ± 0.4 |  | 0.12 | (-0.03, 0.26) | 0.11 |
| Right IFPC | 1.9 ± 0.6 | 1.8 ± 0.7 |  | 0.08 | (-0.07, 0.22) | 0.29 |
| Left LTC | 1.7 ± 0.5 | 1.6 ± 0.4 |  | 0.05 | (-0.09, 0.19) | 0.50 |
| Right LTC | 1.8 ± 0.4 | 1.6 ± 0.4 |  | 0.11 | (-0.03, 0.25) | 0.13 |
| (p-value)‡ |  |  |  |  |  | 0.67 |

Δ = mean difference in ERF responses between known and unknown words after natural log-transformation;

*Values are mean ± SD;

†Test of Δ = 0;

‡Test for any difference in Δ between regions or hemispheres.

**Supplemental Table 2.** Comparison of ERF responses to known and unknown words by region and hemisphere in the middle measurement window.

|  | **400-600 ms Window** | | | | | |
| --- | --- | --- | --- | --- | --- | --- |
|  |  |  |  |  |  |  |
|  | **Word Type*** | |  | **Difference†** | | |
|  | **Known** | **Unknown** |  | **Δ** | **(95% CI)** | **P-value** |
| **Overall** | 2.3 ± 1.0 | 2.3 ± 1.2 |  | 0.03 | (-0.04, 0.10) | 0.41 |
|  |  |  |  |  |  |  |
| **Region** |  |  |  |  |  |  |
| EAC | 2.9 ± 1.3 | 3.2 ± 1.6 |  | -0.07 | (-0.19, 0.04) | 0.21 |
| IFC | 2.0 ± 0.7 | 1.8 ± 0.7 |  | 0.14 | (0.03, 0.26) | 0.015 |
| IFPC | 2.2 ± 0.9 | 2.2 ± 0.8 |  | 0.00 | (-0.11, 0.12) | 0.98 |
| LTC | 2.3 ± 0.8 | 2.2 ± 0.7 |  | 0.04 | (-0.08, 0.15) | 0.51 |
| (p-value)‡ |  |  |  |  |  | 0.036 |
|  |  |  |  |  |  |  |
| **Hemisphere** |  |  |  |  |  |  |
| Left | 2.3 ± 1.0 | 2.2 ± 0.7 |  | 0.02 | (-0.06, 0.11) | 0.57 |
| Right | 2.4 ± 1.1 | 2.5 ± 1.5 |  | 0.03 | (-0.06, 0.12) | 0.48 |
| (p-value)‡ |  |  |  |  |  | 0.91 |
|  |  |  |  |  |  |  |
| **Region-Hemisphere** |  |  |  |  |  |  |
| Left EAC | 2.7 ± 1.1 | 2.5 ± 0.8 |  | 0.03 | (-0.13, 0.18) | 0.73 |
| Right EAC | 3.1 ± 1.5 | 3.9 ± 1.9 |  | -0.17 | (-0.33, -0.02) | 0.028 |
| Left IFC | 2.0 ± 0.8 | 1.6 ± 0.5 |  | 0.16 | (0.00, 0.31) | 0.043 |
| Right IFC | 2.1 ± 0.6 | 1.9 ± 0.9 |  | 0.13 | (-0.02, 0.28) | 0.096 |
| Left IFPC | 2.3 ± 1.0 | 2.1 ± 0.6 |  | 0.04 | (-0.12, 0.19) | 0.62 |
| Right IFPC | 2.1 ± 0.6 | 2.2 ± 1.1 |  | -0.04 | (-0.19, 0.12) | 0.65 |
| Left LTC | 2.0 ± 0.8 | 2.3 ± 0.8 |  | -0.12 | (-0.28, 0.03) | 0.11 |
| Right LTC | 2.5 ± 0.9 | 2.0 ± 0.6 |  | 0.20 | (0.05, 0.35) | 0.011 |
| (p-value)‡ |  |  |  |  |  | 0.005 |

Δ = mean difference in ERF responses between known and unknown words after natural log-transformation;

*Values are mean ± SD;

†Test of Δ = 0;

‡Test for any difference in Δ between regions or hemispheres.

**Supplemental Table 3.** Comparison of ERF responses to known and unknown words by region and hemisphere in the late measurement window.

|  | **600-900 ms Window** | | | | | |
| --- | --- | --- | --- | --- | --- | --- |
|  |  |  |  |  |  |  |
|  | **Word Type*** | |  | **Difference†** | | |
|  | **Known** | **Unknown** |  | **Δ** | **(95% CI)** | **P-value** |
| **Overall** | 2.0 ± 0.7 | 1.9 ± 0.5 |  | 0.03 | (-0.05, 0.11) | 0.44 |
|  |  |  |  |  |  |  |
| **Region** |  |  |  |  |  |  |
| EAC | 2.2 ± 0.9 | 2.1 ± 0.6 |  | -0.01 | (-0.12, 0.10) | 0.88 |
| IFC | 2.0 ± 0.7 | 1.7 ± 0.6 |  | 0.13 | (0.02, 0.24) | 0.021 |
| IFPC | 1.7 ± 0.4 | 1.7 ± 0.4 |  | 0.01 | (-0.10, 0.12) | 0.86 |
| LTC | 1.9 ± 0.7 | 1.9 ± 0.5 |  | -0.02 | (-0.13, 0.09) | 0.78 |
| (p-value)‡ |  |  |  |  |  | 0.11 |
|  |  |  |  |  |  |  |
| **Hemisphere** |  |  |  |  |  |  |
| Left | 1.9 ± 0.7 | 1.9 ± 0.5 |  | -0.02 | (-0.11, 0.07) | 0.61 |
| Right | 2.0 ± 0.7 | 1.9 ± 0.6 |  | 0.08 | (-0.01, 0.17) | 0.075 |
| (p-value)‡ |  |  |  |  |  | 0.031 |
|  |  |  |  |  |  |  |
| **Region-Hemisphere** |  |  |  |  |  |  |
| Left EAC | 2.2 ± 1.0 | 2.2 ± 0.7 |  | -0.03 | (-0.18, 0.11) | 0.67 |
| Right EAC | 2.2 ± 0.8 | 2.1 ± 0.5 |  | 0.01 | (-0.13, 0.16) | 0.85 |
| Left IFC | 1.8 ± 0.7 | 1.7 ± 0.4 |  | 0.07 | (-0.07, 0.21) | 0.34 |
| Right IFC | 2.2 ± 0.7 | 1.8 ± 0.7 |  | 0.19 | (0.05, 0.34) | 0.010 |
| Left IFPC | 1.7 ± 0.3 | 1.8 ± 0.3 |  | -0.05 | (-0.20, 0.09) | 0.46 |
| Right IFPC | 1.8 ± 0.4 | 1.7 ± 0.4 |  | 0.07 | (-0.07, 0.22) | 0.31 |
| Left LTC | 1.9 ± 0.8 | 2.0 ± 0.5 |  | -0.07 | (-0.22, 0.07) | 0.31 |
| Right LTC | 2.0 ± 0.7 | 1.8 ± 0.5 |  | 0.04 | (-0.10, 0.19) | 0.55 |
| (p-value)‡ |  |  |  |  |  | 0.92 |

Δ = mean difference in ERF responses between known and unknown words after natural log-transformation;

*Values are mean ± SD;

†Test of Δ = 0;

‡Test for any difference in Δ between regions or hemispheres.
